# Novel Identified Receptors on Mast Cells

**DOI:** 10.3389/fimmu.2012.00238

**Published:** 2012-08-02

**Authors:** Helena Migalovich-Sheikhet, Sheli Friedman, David Mankuta, Francesca Levi-Schaffer

**Affiliations:** ^1^Department of Pharmacology and Experimental Therapeutics, Institute for Drug Research, School of Pharmacy, Faculty of Medicine, The Hebrew University of JerusalemJerusalem, Israel; ^2^Department of Obstetrics and Gynecology, Hadassah University HospitalJerusalem, Israel

**Keywords:** mast cells, activating receptors, inhibitory receptors, ligands, signal transduction, function, co-stimulation/cross-talk

## Abstract

Mast cells (MC) are major participants in the allergic reaction. In addition they possess immunomodulatory roles in the innate and adaptive immune reactions. Their functions are modulated through a number of activating and inhibitory receptors expressed on their surface. This review deals with some of the most recently described receptors, their expression patterns, ligand(s), signal transduction mechanisms, possible cross-talk with other receptors and, last but not least, regulatory functions that the MC can perform based on their receptor expression in health or in disease. Where the receptor role on MC is still not clear, evidences from other hematopoietic cells expressing them is provided as a possible insight for their function on MC. Suggested strategies to modulate these receptors’ activity for the purpose of therapeutic intervention are also discussed.

## Background

Mast cells (MC) have the uniqueness of being tissue resident cells packaged with cytoplasmic granules full of preformed mediators of diverse nature, rich in surface receptors that upon ligand binding can induce not only the fast release of the stored mediators, but also the *de novo* synthesis of arachidonic acid metabolites and a number of chemokines and cytokines. Therefore they play a prominent role in maintaining homeostasis, acting as armed sentinel cells in the tissues, where they reside spanning from mucosal to connective tissues and more. MC have been historically associated with allergy, in which the key receptor is FcεRI. However, their strategic location and potential have clearly demonstrated that MC are more than the primum movens of allergic inflammation; they are very important players also in innate and adaptive immune responses, inflammation, and tissue changes. MC immunomodulatory roles may result in either negative or positive outcome for the host, enhancing, or suppressing certain features of immune/inflammatory responses. A large variety of receptors especially activating and some inhibiting MC functions, have been described. FcεRI, for IgE activation, and c-kit, the receptor of the stem cell factor (SCF), serve as fingerprints in MC characterization and are the topic of other reviews of this series.

Our purpose is to review herewith the expression pattern, function, ligand(s), and signal transduction pathways of some of the most recently described activating and inhibiting receptors identified to be expressed by the MC from different origins. We have mainly reviewed the novel activating receptors and discussed just a few examples of the inhibitory ones. The activating receptors are comprised of chemokines, interleukins (IL), amines, Toll-Like Receptors (TLRs), and others (Table 1). For some of the receptors, if known, the physiological and pathological consequences of their activation and the strategies to modulate their activity for the purpose of therapeutic intervention are also discussed. Evidences from other hematopoietic cells expressing these receptors is provided as a possible insight for their function on MC. Potential cross-talk between activating on one side and activating and inhibitory receptors (IRs) on the other side is also briefly considered.

**Table 1:** **Structural functional data, expression, ligand, and function of some MC receptors discussed in the review**.

Receptor	Ligand	Structural functional data	MC expression	Function
Chemokines receptors	For CXCR3-CXCL9, CXCL10, and CXCL11 (Meiser et al., 2008)	GPCR	E.g., CXCR3 protein: human lung MC (Juremalm and Nilsson, 2005)	Migration (Juremalm et al., 2002), signaling events (Lacotte et al., 2009) partial degranulation (Willox et al., 2010)
IL-15R	IL-15	α Chain, β chain, γ chain	Protein: mBMMC Expression assumed by function: hCBMC (Jackson et al., 2005)	Migration (Jackson et al., 2005), enhancement of Th1 response, inhibition of allergic inflammation in a murine model of asthma (Ishimitsu et al., 2001)
IL-18R	IL-18	Dimeric complex of IL-18Rα, IL-18Rβ	mRNA and protein: mouse mucosal MC and immature MC (Wiener et al., 2008)	Production of IFN γ, GM-CSF, TNF-α and IL-1, IL-13, and/or IL-4 (Nakanishi et al., 2001; Akdis et al., 2011)
IL-33R (ST2)	IL-33	Complex: ST2 and IL-1RAcP	Protein: mBMMC (Liew et al., 2010)	Degranulation and proinflammatory cytokine production and release (Hsu et al., 2010; Liew et al., 2010)
TSLPR	TSLP	Complex: TSLPR with IL-7Rα	mRNA: mBMMC (Knisz et al., 2009). Protein: human peripheral blood and hCBMC (Allakhverdi et al., 2007)	Increasing of pro-inflammatory cytokines (Allakhverdi et al., 2007) production and Th2 response (Ziegler and Artis, 2010)
TLR	Bacterial and viral proteins	Extracellular leucine rich region, cytoplasmic domain that consists of three homologous regions (box 1, 2, 3)	TLR2 and TLR4 mRNA:immature and mature MC from murine and human origins (Supajatura et al., 2002). TLR2 protein: hCBMC and nasal polyps MC (McCurdy et al., 2003). TLR2 and TLR4 protein:murine intestine MC and murine and rat peritoneal MC (Pietrzak et al., 2011)	*de novo* synthesis and release of cytokines (McCurdy et al., 2001; Supajatura et al., 2002)
CD48	2B4, FimH, *S. aureus*, and *M. tuberculosis* toxins	Glycosyl-phosphatidyl-inositol (GPI)-anchored protein	Protein: BMMC and human peripheral blood MC	Allergic effector unit formation, stimulation of mediator release (Elishmereni et al., 2011)
S1P2R	S1P	GPCR	Protein: mBMMC and RBL-2H3 (Jolly et al., 2004), hMC (Oskeritzian et al., 2010), and hematopoietic progenitors (Price et al., 2009)	Degranulation (Wang et al., 2012) and chemokine and cytokine release (Oskeritzian et al., 2010) trafficking and migration (Spiegel and Milstien, 2011)
HR (H_1_, H_2_, H_3_, H_4_)	Histamine	GPCR	H1R mRNA and protein: (Lippert et al., 2004) low in human skin MC (Lippert et al., 2004; Gibbs and Levi-Schaffer, 2012), higher in HMC-1 cells	H1, H2-intracellular calcium mobilization (Tilly et al., 1990)
			H2R mRNA and protein: human skin MC, and HMC-1 cells (Lippert et al., 2004)	H3-autoregulation of histamine release (Ohkubo et al., 1994)
			H_3_R protein: in brain mast cells (Rozniecki et al., 1999) H4R mRNA: mouse MC, hCBMC, and HMC-1 (Hofstra et al., 2003; Gibbs and Levi-Schaffer, 2012), human skin MC (Lippert et al., 2004)	H4-chemotaxis and intracellular calcium mobilization in mMC (Nordlind et al., 2008), recruitment of effector cells in human (Hofstra et al., 2003)
5HTR	Serotonin	GPCR	mRNA: mBMMC and human CD34+-derived MC (Kushnir-Sukhov et al., 2006)	Cell migration (Nordlind et al., 2008)
Purinergic P1	Adenosine	P1: GPCR	Adenosine receptors-A1protein: canine BR mastocytoma A2a protein: HMC-1, RBL-2H3, mBMMC	hCBMC implicating the A_1_R in potentiation, and A_2_BR receptor in the inhibition of anti-IgE-induced degranulation (Yip et al., 2009; Hua et al., 2011)
			A2b protein: canine BR mastocytoma, HMC-1, RBL-2H3, mBMMC	
			A3 protein: mastocytoma, RBL-2H3, mBMMC (Forsythe and Ennis, 1999; Hua et al., 2011)	
P2X	ATP	P2X: ion pore	E.g., P2X_1_, P2X_4_, P2X_7_ protein: hCBMC(Bulanova and Bulfone-Paus, 2010). P2X_1–4_, P2X_6–7_ mRNA: mBMMC (derived from C57BL/6 mice)	Degranulation, apoptosis (Bulanova et al., 2009)
P2Y	ATP, ADP, UTP, UDP, UDP-glucose	P2Y: GPCR	P2Y receptors (with the exception of P2Y_2_) protein: mBMMC	Degranulation, P2Y_13_ in RBL-2H3 Ca^2+^ release and release of β-hexosaminidase
			P2Y_1_, P2Y_2_ P2Y_12_, P2Y_13_, and P2Y_11_: hCBMC (Feng et al., 2004)	P2Y_2_: mediation of chemotaxis in mBMMC and CD34+ progenitors. (Bulanova and Bulfone-Paus, 2010)
CD203c	NAD and nucleotide sugars	Type II-phosphatidyl-inositol transmembrane proteins with catalytic domain and a C-terminal endonuclease-like domain	mRNA and protein: overexpressed on neoplastic MC in patients with systemic mastocytosis (Hauswirth et al., 2008)	Cleavage of phosphodiester and phosphosulfate bonds (Buhring et al., 2004)
CRHR	CRH	GPCR	mRNA and protein: hCBMC and LAD-2 (Asadi et al., 2012)	Production and release of IL-8, TNF, VEGF. Induction of NK-1 gene expression (Asadi et al., 2012)
Endocannabinoids receptors	Endocannabinoids	GPCR	Protein: on human skin MC (Sugawara et al., 2012)	Inhibition of MC maturation and activation, regulation of SCF expression (Sugawara et al., 2012)

*mRNA, messenger ribonuclear acid; MC, mast cell; IL, interleukin; GPCR, G protein coupled receptor; H, human; M, mouse; BMMC, bone marrow mast cells; CBMC, cord blood mast cells; IFN γ, interferon-gamma; GM-CSF, granulocyte-macrophage colony-stimulating factor; TNF α, tumor necrosis factor α; TSLPR, thymic stromal lymphopoietin receptor; TLR, Toll-like receptor; 5HTR, 5-hydroxytryptamine receptors; ATP, ADP, adenosine 5′-triphosphate, adenosine diphosphate; UTP, UDP, uridine triphosphate, uridine diphosphate; NAD, nicotinamide adenine dinucleotide; CRHR, corticotropin-releasing hormone receptor; CRH, corticotropin-releasing hormone; SCF, stem cell factor; S1P, sphingosine-1-phosphate; HR, histamine receptor*.

## Chemokines Receptors

Chemokines are small cytokine-like proteins that regulate leukocyte trafficking under homeostatic and inflammatory conditions (Luther et al., 2002; Homey and Bunemann, 2004; for chemokines receptors (CRs), CRs in MC, see review Juremalm and Nilsson, 2005). CRs, expressed on hematopoietic and lymphatic cells, are regulated by a variety of inflammatory stimuli (Olson and Ley, 2002). For example CXCR1 and CXCR2 are expressed on most leukocytes, but appear to be functionally significant only for neutrophils, monocytes/macrophages, and MC. CXCR3, CXCR5, and CXCR6 were found to be expressed on cells of lymphoid lineage (Olson and Ley, 2002). CXCR3 was later found to be expressed also by MC (Juremalm and Nilsson, 2005). A defined set of chemokines (CCL1–5, CCL11, CCL13, CCL17–18, CCL20, CCL22, CCL26–27, CX3CL1) was identified to initiate and perpetuate atopic skin inflammation (Homey et al., 2006, 2007).

Human MC (hMC) from different origins express at least nine CRs, i.e., CXCR1–4, CX3CR1, CCR1 (further detailed in MC Co-Stimulation), CCR3–5 that may be activated by the respective chemokines, i.e., CXCL1, CXCL5, CXCL8, CXCL14, CX3CL1, CCL5, and CCL11 (Juremalm and Nilsson, 2005). Interestingly, activation of CRs on MC can not only induce their chemotaxis, but also other stimulatory responses. For example, the activation of CXCR3 and CCR2, besides migration also induces human cord blood MC (hCBMC) signaling events or even partial degranulation in the absence of antigen (Willox et al., 2010). The expression of CXCR3 and RANTES (also known as CCL5) on MC is important especially in the migration of MC precursors in the tissues where they undergo final maturation (Juremalm et al., 2002). CCL5/RANTES can induce MC migration through interactions with CCR1 and CCR4 (Juremalm et al., 2002).

*CXCR3* is a seven transmembrane (TM) domain G protein coupled receptor (GPCR) that binds the pro-inflammatory, non-ELR motif of CXC chemokines: CXCL9, CXCL10, and CXCL11 (Meiser et al., 2008) and was found to be sensitive to pertussis toxin (Ptx; Willox et al., 2010). Recently, two different variants of CXCR3 have been identified in human: CXCR3B and CXCR3-ALT, both originating from the same gene and produced by alternative splicing of the CXCR3 mRNA (Willox et al., 2010). CXCR3 activation leads to Gαi protein internalization with subsequent calcium influx and triggering of mitogen-activated protein kinases (MAPK) and AKT (Protein kinase B, PKB) cascade resulting in cytoskeleton rearrangement and cell movement (Lacotte et al., 2009). Upon activation of this CR, cross-talk between the CXCR3 and FCεRI is possible due to the fact that both these receptors involve phosphoinositide 3-kinases (PI3Ks) signaling. CXCR3 is highly expressed in human lung MC that reside in proximity to the smooth muscle in asthmatics, but is not expressed in human bone marrow MC (hBMMC; Brightling et al., 2005). In human lungs, CXCR3 on MC mediates their migration toward chemokines-secreting airway smooth muscle regions. Additionally, CXCL10 has been shown to be expressed in that region in bronchial asthma biopsy specimens and *ex vivo* in smooth muscle cells from asthmatics compared to the ones derived from healthy volunteers (Okayama et al., 2008). Because of the importance of chemokines in recruiting inflammatory cells and MC precursors into the tissues, neutralization of chemokines, or antagonists of their receptors is an active investigational field in the pharmaceutical industry.

## Interleukin Receptors and TSLPR

Interleukins are secreted glycoproteins that bind to their specific receptors and play a role in the communication among leukocytes. In the past decade, a number of new cytokines have been described such as IL-15, IL-18, IL-7, IL-33. Importantly, MC display the receptors for some of the newly identified IL.

### The IL-15 receptor

The classic IL-15 receptor (*IL-15R*) consists of a unique α chain, a β chain, and a common γ chain which is shared with the IL-2 receptor (Lorenzen et al., 2006). IL-15R signaling requires DAP10-associated proteins since the IL-15 signaling pathway phosphorylates DAP10 through JAK3. Once activated, IL-15R triggers the activation of JAK3-STAT5 signaling pathway that relays information to the nucleus (Colucci, 2007). IL-15R is expressed in B, T cells, and natural killers cells (NK) and its activation results in cell proliferation and differentiation. IL-15R was recently reported to be expressed on mouse BMMC (mBMMC). It has not yet been reported whether hCBMC express the same receptors. However, IL-15 induced migration of both mBMMC and hCBMC in a dose-responsive and biphasic manner, supports the possibility that this effect is mediated by two distinct IL-15R with different affinities to IL-15 (Jackson et al., 2005). Interestingly, in stimulated dendritic cells (DC), IL-15, and IL-15Rα are preassembled in complexes within the endoplasmic reticulum/Golgi before being released from the cells. In NK, membrane bound IL-15-IL-15Rα complexes, and not the soluble ones, trigger activation (Mortier et al., 2008).

The ligand of IL-15R, IL-15, is produced in human by both non-immune cells (keratinocytes and skeletal muscle cells) and by immune cells (monocytes, and activated CD4+ T cells) in response to signals that induce innate immunity (Akdis et al., 2011). IL-15 is also produced by murine MC, especially after stimulation with lipopolysaccharide (LPS; Orinska et al., 2007). IL-15 in MC induces an intracrine signal that downregulates chymase-dependent, MC-mediated innate immunity (Orinska et al., 2007). In *Il-15*^−/−^ mice, the absence of IL-15 in MC increases chymase activities, leading to greater MC bactericidal responses, increased processing, and activation of neutrophil-recruiting chemokines, and improved sepsis survival (Orinska et al., 2007).

IL-15 is associated with autoimmune and inflammatory diseases and was recently shown to be upregulated in T cell-mediated inflammatory disorders, such as rheumatoid arthritis (RA) and inflammatory bowel diseases (Waldmann, 2004). In rheumatoid synovial tissue explants, αIgE induced MC activation induces changes in the amounts of released tryptase, TNFα, and IL-1β by a proportion of mononuclear inflammatory cells, but not in the amount of IL-15 (Woolley and Tetlow, 2000). IL-15 is also associated with the development of X-linked severe combined immunodeficiency (Akdis et al., 2011). Overexpression of IL-15 *in vivo* enhances Th1 responses, which inhibit allergic inflammation in a murine model of asthma (Ishimitsu et al., 2001). In a human psoriasis xenograft model, the anti IL-15 antibody 146B7 was found to be involved in the reduction of the severity of the disease (Villadsen et al., 2003). Additionally, HuMax-IL-15, a human IgG1 anti-IL-15 monoclonal antibody, that *in vitro* was able to neutralize exogenous and endogenous IL-15, has been used for clinical trials in patients with RA to perform a phase I–II dose-escalation trial (Baslund et al., 2005; Waldmann, 2006).

### The IL-18 receptor

IL-18 receptor (IL-18R) expressed on MC belongs to the IL-1 receptor (IL-1R) family (Thomassen et al., 1998). IL-18Rα is the binding chain and together with IL-18Rβ form a high affinity heterotrimeric complex with the ligand IL-18. This complex recruits the intracellular adapter molecules myeloid differentiation primary response protein 88 (MyD88), IL-1 receptor-associated kinases (IRAK) and tumor necrosis factor receptor associated factor 6 (TRAF6) which results in the activation of p38 MAPK, JUN N-terminal kinase (JNK), and/or nuclear factor kB (NF-kB; Arend et al., 2008). IL-18R is expressed on T cells, NK cells, macrophages, epithelial cells, chondrocytes (Kunikata et al., 1998), and MC (Helmby and Grencis, 2002).

The ligand of IL-18R, IL-18, is an IFN-γ-inducing factor and a potent cytokine that is produced by monocytes/macrophages as a reaction to different microbial components and therefore plays a major role in the innate immune responses to pathogens (Arend et al., 2008). Also Kupffer cells, keratinocytes, osteoblasts, astrocytes, and DC express IL-18 (Akdis et al., 2011). The regulation of IL-18 biological activity is carried out by caspase-1-mediated cleavage of pro-IL-18 to the mature protein. Human chymase can also cleave pro-IL-18 to the active protein (Omoto et al., 2006). IL-18 binding protein (BP) is a soluble protein that by binding mature IL-18 prevents its binding to receptor, and therefore serves as a natural inhibitor (Novick et al., 1999). IL-18BP displays four isoforms in humans and two in mice that are the result of alternative splicing of mRNA and which mainly differ in the C-terminal region. IL-18 indirectly increases the production of its own inhibitor in a feedback loop via INFγ secretion. IL-18R activation is able to induce in addition to IFNγ, also GM-CSF, TNFα, and IL-1 production. In addition it can induce IL-13 and/or IL-4 production by NK, MC, and basophils. It can therefore enhance innate immunity and both Th1 and Th2 driven immune responses (Nakanishi et al., 2001).

IL-18 has been associated with autoimmune diseases or inflammatory disorders, bronchial asthma (Harada et al., 2009), atopic dermatitis (Konishi et al., 2002), RA (Gracie et al., 1999; Plater-Zyberk et al., 2001), psoriasis (Ohta et al., 2001), multiple sclerosis (Karni et al., 2002), and type I diabetes (Altinova et al., 2008; Akdis et al., 2011). Regarding allergy, interestingly IL-18 contributes to the spontaneous development of IgE/signal transducer and activator of transcription (STAT6)-independent atopic dermatitis-like inflammatory skin lesion (Konishi et al., 2002).

In conclusion, IL-18/IL-18R regulation, if carried out by selective antagonists, can possibly be considered as a good pharmacological target in either Th1 or Th2 driven conditions.

### IL-33 receptor

IL-33 receptor (*IL-33R*) termed also *ST2* is a member of the IL-1R family, and TLR/IL-1R (TIR) superfamily (Liew et al., 2010). It binds IL-33 as a heterodimer consisting of ST2 and IL-1R accessory protein. The signaling cascade of ST2 activation involves recruitment of MyD88, TRAF6, and IRAK. This leads to NF-kB activation and the activation of MAPK p38, signal-regulated kinase ERK, and JNK. ST2 is expressed on basophils, MC, eosinophils, NK cells, Th2 cells, DC, and nuocytes (Kunikata et al., 1998; Akdis et al., 2011).

Mouse BMMC express high levels of ST2 and respond directly to IL-33 to produce a spectrum of inflammatory cytokines and chemokines (IL-1, IL-6, IL-13, TNF, CCL2, and CCL3; Liew et al., 2010). *In vivo*, IL-33 treatment exacerbated collagen-induced arthritis in *ST2*^−/−^ KO mice engrafted with MC from WT but not from *ST2*^−/−^ mice, a fact that was associated with elevation in expression levels of pro-inflammatory cytokines (Xu et al., 2008). The ST2 ligand, IL-33, is a pro-inflammatory cytokine that activates Th2 response-inducing cells. IL-33 activation induces mBMMC and the murine MC line MC/9 proliferation and Th2 cytokine production including IL-4, IL-5, IL-6, but not IL-33 itself (Hsu et al., 2010). On the other hand mBMMC produce IL-33 in response to IgE activation, which requires calcium influx or ionomycin activation (Hsu et al., 2010).

IL-33/ST2 pathway is critical for the development of IgE-driven tissue inflammation during passive cutaneous anaphylaxis that is a strictly MC-dependent model (Hsu et al., 2010). Levels of soluble ST2 increase in inflammatory conditions such as systemic lupus erythematosus (SLE), RA (Xu et al., 2008), idiopathic pulmonary fibrosis (Tajima et al., 2003), asthma (Oshikawa et al., 2001), progressive systemic sclerosis, Behcet’s disease, Wegener’s granulomatosis, severe trauma, and sepsis (Akdis et al., 2011). Importantly, in all of these conditions MC activation and role have been illustrated. IL-33 release is elevated in skin of patients with atopic dermatitis (Liew et al., 2010). Constant mechanical microtrauma and destruction of skin barrier induce IL-33 release and a pro-inflammatory response. In addition, IL-33 may play a role in psoriasis-like plaque inflammation (Hueber et al., 2011).

IL-33 has been classified as an alarmin/danger signal (Cevikbas and Steinhoff, 2012) since it is released by cells undergoing necrosis and it is inactivated by caspases during cell apoptosis (Liew et al., 2010; Hueber et al., 2011). However, it is evident that IL-33 is released not only from necrotic cells, but also from living cells and therefore acts as a classical cytokine (Liew et al., 2010). Its importance in allergy is the current topic of high interest partly due to its effects on the MC.

### TSLP receptor

TSLP receptor (*TSLPR*) is the receptor of TSLP that is an IL-7-like cytokine initially identified in the culture supernatant of a thymic stromal cell line (He and Geha, 2010). TSLPR has a low affinity to TSLP, but together with IL-7Rα they generate high affinity binding sites and trigger signaling (Park et al., 2000). Cross species homology of TSLP and its receptor is relatively low (about 40%; He and Geha, 2010; Ziegler and Artis, 2010), but the fact that both in human and in mouse IL-7Rα is required suggests that human TSLP and TSLPR are orthologs to mouse TSLP and TSLPR. The signaling cascade of these receptors is not completely clear and no Janus kinases (JAK) are activated (Isaksen et al., 2002). It has however been shown that receptor engagement can activate the transcription factor STAT3 in human and STAT5 in mouse and human (Isaksen et al., 2002). In addition, it can also induce the expression of common genes (such as *Cish*; Isaksen et al., 2002).

TSLP receptor is expressed on hematopoietic cell lineages, including B cells, T cells, MC, eosinophils, and DC (Taylor et al., 2009). mBMMC were shown to express TSLPR mRNA (Knisz et al., 2009). TSLPR and IL-7Rα chain expression were determined at the mRNA level on human peripheral blood and hCBMC (Allakhverdi et al., 2007). In spite of these reports, human peripheral blood derived MC (hpbMC) and the HMC-1 cell line (hMC leukemia-1) were found by us to express TSLPR, but not IL-7R (Levi-Schaffer, F., Soumelis, V., Levy, I., unpublished data). At the protein level, TSLPR was also reported to be expressed *in vivo* on MC infiltrating the bronchial mucosa of asthmatic patients, as revealed by immunostaining of biopsy specimen (Allakhverdi et al., 2007; Comeau and Ziegler, 2010; Shikotra et al., 2012).

It has been found that MC infiltrating to the mucosal gland stroma and airway smooth muscle in asthma not only express high levels of TSLP mRNA, but can also respond to TSLP (Rochman and Leonard, 2008). TSLP can directly activate hMC to produce pro-inflammatory Th2 cytokines and chemokines in the presence of IL-1β and tumor necrosis factor α (TNF-α), in a way that mimics inflammation conditions (Allakhverdi et al., 2007). Various cell types can produce TSLP: endothelial, epithelial cells and epidermal keratinocytes, airway smooth muscle cells, fibroblasts, DC, trophoblasts, and cancer or cancer-associated cells (Takai, 2012). hMC express TSLP mRNA, which is upregulated upon cross-linking of the IgE receptor. Pre-incubation with IL-4 results in significant upregulation of IgE-mediated TSLP protein and mRNA expression (Okayama et al., 2009; Comeau and Ziegler, 2010).

TSLP plays a significant role in initiation of allergic inflammation and is very important to cells such as the MC, due to its high expression in the interfaces between the body and environment and its ability to lead to Th2 responses (Ziegler and Artis, 2010). Different studies have shown that asthma, allergic rhinitis, and atopic dermatitis are characterized by an increased expression of TSLP in the inflamed tissue (Ziegler and Artis, 2010; Le et al., 2011). Mice overexpressing TSLP on *TCR*β^−/−^ background, develop dermal inflammation and skin infiltrates of MC and eosinophils (Yoo et al., 2005). MC deficient mice failed to upregulate TSLP in nasal epithelium after allergen challenge in a model of allergic rhinitis (Ziegler and Artis, 2010). In asthma it was demonstrated that MC play an important role in TSLP production (Shikotra et al., 2012). TSLP levels are increased in human asthma and correlate with the increase in expression of Th2 cytokines and disease severity (Ying et al., 2005, 2008; Corrigan et al., 2009; Fang et al., 2010).

Blocking of TSLP signaling using TSLPR-immunoglobulin in murine asthma model, was shown to regulate pulmonary DC function and to reduce eosinophilic airway inflammation and Th2 differentiation significantly (Zhang et al., 2011). Additionally, TSLP/TSLPR interaction influences the function of cells in host protection against helminth parasites, and modulates gut homeostasis in conditions in which MC are involved (Comeau and Ziegler, 2010). Therefore it appears that MC displaying TSLPR and producing TSLP have a potent weapon in addition to FcεRI in orchestrating the allergic reactions. As a consequence the TSLP axis is a very promising candidate for anti-allergic intervention.

Notably, TSLP and IgE stimulated MC can induce OX40 ligand (OX40L) expression on DC. (Ito et al., 2005; Edwards, 2008). OX40 receptor and its ligand OX40L belong to the superfamilies TNF receptor and TNF respectively (Godfrey et al., 1994) and were shown to have a crucial role in allergic inflammation. OX40/OX40L interaction can trigger the differentiation of some inflammatory Th2 cells in the lymph nodes. (Liu, 2007; Wang and Liu, 2007) OX40L on MC and OX40 on T regulatory cells cross-talk can induce T cells activation by hMC *in vitro* (Kashiwakura et al., 2004) and inhibit FcεRI-dependent MC degranulation both *in vitro* and *in vivo* (Gri et al., 2008). Recently, Ilves and Harvima (2012) showed that there are more OX40 positive cells in the dermis from AD lesions than in healthy looking dermis, which does not correlate with clinical severity of AD.

## Toll-Like Receptors and Other Bacterial and Viral Receptors

As key protagonists of innate immunity, MC play a pivotal role in anti-infection defense and “danger” responses. MC reactivity against bacteria have been more characterized than the ones against viruses, although both of them are classically mediated by TLRs (Akira and Takeda, 2004), a family of pattern recognition molecules.

Toll-like receptors and IL-1R have a conserved cytoplasmic domain that consists of three homologous regions (boxes 1, 2, 3) and an extracellular domain that differs: TLRs have tandem repeats of leucine rich regions, while IL-1R has three immunoglobulin like domains (Akira and Takeda, 2004). Stimulation of TLR triggers the association of MyD88, IRAK4, and IRAK1 signaling leading to the NF-kB activation and gene expression (Akira and Takeda, 2004). MC signaling via TLRs involves also ITAM-containing molecule DAP12 phosphorylation that activates syk, which is a critical molecule of MC activation (Smrz et al., 2010). This would suggest a cross-talk between TLRs and receptors that employ DAP12 as a transducer molecule.

TLR2 and TLR4 expression has been detected at the mRNA level in immature and mature MC from murine and human origins (Supajatura et al., 2002). At the protein level, TLR2 was found to be expressed in hCBMC and in nasal polyps MC (McCurdy et al., 2003). TLR2 and TLR4 are found in murine intestine MC and murine and rat peritoneal MC (Pietrzak et al., 2011). TLR signaling events induce cytokine production (Supajatura et al., 2002). TLR2 can be activated by prolonged stimulation with bacterial wall components, such as LPS and peptidoglycan (PGN), resulting in *de novo* synthesis and release of various cytokines. It was shown that PGN was able to induce degranulation of mBMMC via TLR2 accompanied by Ca^2+^ influx, whereas the activation of MC by LPS via TLR4 did not lead to degranulation (Supajatura et al., 2002). Cytokine exposure of MC can also upregulate the expression of TLRs. For example, IL-6 treatment of mMC induces an increase in TLR4 expression, whereas exposure to TNF does not influence the TLR2 and TLR4 protein levels (McCurdy et al., 2001). On the contrary, exposure of MC to CCL5 resulted in decreased expression of both TLR2, following 24 h incubation, and TLR4 level, following 12 and 24 h incubation (McCurdy et al., 2001).

### CD48

*CD48*, a CD2-like molecule, is a 40KD glycosyl-phosphatidyl-inositol (GPI)-anchored protein, expressed on the surface of hematopoietic cells (recently reviewed in Elishmereni and Levi-Schaffer, 2011). The CD48 structure combines a distal V-like domain with a C2-like domain containing conserved cysteine residues that form disulfide bonds. It lacks a TM domain and is attached to the cell surface by a glycolipid, GPI, restricted to the outer leaflet of the membrane bilayer (Shin and Abraham, 2001). CD48 is found also in soluble form, due to its cleavage upon activation. Stimulated CD48 associates to the kinase LCK and leads to tyrosine phosphorylation (Elishmereni and Levi-Schaffer, 2011).

CD48 is illustrated here since in MC, CD48 was first described to be involved in innate immunity as it binds FimH of *E. coli*. This interaction triggers a strong TNF-α release and the uptake of this Gram-bacteria (Malaviya et al., 1999; Malaviya and Abraham, 2001; Proft and Baker, 2009). CD48, has also been implicated in MC interactions with *M. tuberculosis*, which triggers the release of several pre-stored mediators, such as histamine and β-hexosaminidase, and the *de novo* synthesis of cytokines, such as TNF-α and IL-6 (Munoz et al., 2003). Later on we found that *S. aureus*, a Gram+ bacteria, and its toxins bind to CD48 and TLR2 on hCBMC causing subsequent release of pro-inflammatory cytokines (Rocha-de-Souza et al., 2008). CD48 is overexpressed in murine asthma and was defined as a signature gene in this condition (Zimmermann et al., 2003). The CD48 ligand in human is 2B4, expressed by several hematopoietic cells. CD48/2B4 interactions taking place between MC and eosinophils are important in allergic inflammation giving rise to the physical formation of the allergic effector unit (AEU) between these two cells (Elishmereni et al., 2011). CD48-2B4 binding induces degranulation of MC and increases eosinophil survival and activation (Elishmereni et al., 2011). In a murine model of allergic asthma, treatment with neutralizing CD48 Ab dramatically inhibited the lung inflammation (Munitz et al., 2007). Therefore it seems that CD48 is a good candidate to be blocked on MC in order to avoid both AEU formation and bacterial invasion, with this last property being particularly helpful in allergic conditions, such as atopic dermatitis, that are commonly associated with *S. aureus* infection.

### S1P receptors

*S1PR*_1–5_ are GPCRs of sphingosine-1-phosphate (S1P). Secretion of S1P by MC can modulate their function by binding to S1PR_1_ and S1PR_2_ found on MC, in an autocrine fashion (Spiegel and Milstien, 2011). It was shown on DC that there is a cross-talk between S1PR_2_ and S1PR_1_, when upregulation of S1PR_2_ activates the small GTPase RHO leading to translocation of Four and a Half LIM domains protein 2 (FHL2) to the nucleus and downregulates S1PR_1_ expression. On the other hand, downregulation of S1PR_2_ increases S1PR_1_ expression and RAC activation (Spiegel and Milstien, 2011).

S1P_1_ is involved in the migration of MC toward low concentrations of antigen, while S1P_2_ participates in FcεRI-induced degranulation (Jolly et al., 2005; Olivera, 2008). S1P released from MC after cross-linking of FcεRI, and their S1P_2_ receptors are critical for degranulation and chemokine, cytokine, and lipid mediator release from activated human and rodent MC (Oskeritzian et al., 2010). Additionally, blocking of the S1P–S1PR_2_ axis was shown to drastically reduce circulating levels of histamine in a mouse anaphylaxis model indicating that MC were inhibited (Oskeritzian et al., 2010). The S1PR_2_ activation plays a role in anti-viral immunity: the viral membrane lipid sphingomyelin that is converted in the cell membrane to S1P can then activate the S1PR_2_ in an autocrine manner to stimulate MC degranulation (Wang et al., 2012). S1P–S1PR_1_ axis has been shown to be expressed on and to control the trafficking and migration of numerous types of immune cells, including T and B lymphocytes, natural killer T cells, DC, macrophages, neutrophils, hematopoietic progenitors, MC, and osteoclasts (Spiegel and Milstien, 2011).

Therefore, S1PR can offer an interesting pharmacological target for a number of immune diseases. For example the S1PR_2_ antagonist JTE013 significantly inhibited H_2_O_2_-induced permeability in the rat lung perfused model (Sanchez et al., 2007). More studies are under way to exploit the blockage of the S1P and its receptors in many diseases and notably in allergy.

## Amines’ Receptors

Among the mediators extensively identified with the function of MC in allergy, histamine in humans and rodents, and serotonin especially in rodents are key factors influencing the activity of a number of target cells/organs. MC not only produce these two potent amines but also display some of their receptors (Gibbs and Levi-Schaffer, 2012; Ritter et al., 2012).

### Histamine receptors

Histamine receptors (*HRs*) are GPCRs with seven TM-spanning helices. Until now, four subtypes of human HRs have been identified: H1R, H2R, H3R, and H4R. H1R and H2R have been the focus of many studies (recently reviewed in Gibbs and Levi-Schaffer, 2012). Fewer studies have been performed on the H3R and even less on the newest identified one, H4R. Regarding HRs expression by MC, not much work had been carried out until the recent discovery of H4R.

H1R expression has been described on human skin MC to be low (Lippert et al., 2004; Gibbs and Levi-Schaffer, 2012), while on the HMC-1 cells it is higher (Lippert et al., 2004). H1R antagonists can inhibit MC activation and therefore can be used as anti-allergic/MC stabilizing drugs (Levi-Schaffer and Eliashar, 2009), even though some of them might increase cellular cAMP in competitive antagonism with H2R. In cholangiocytes, H1R acts by Gαq activating IP(3)/Ca^2+^, whereas H2R activates Gα(s) stimulating cAMP (Francis et al., 2012). Additionally, it was reported that H2R can activate both adenylate cyclase and phospholipase C signaling pathways via separate GTP-dependent mechanisms (Wang et al., 1996). Interestingly, via the H2R, histamine displays a net inhibitory action on MC (Gibbs and Levi-Schaffer, 2012). H2R is expressed on human skin MC, on HMC-1 cells and basophils, and perhaps on human lung MC. The H2 agonist impromidine was shown to inhibit MC activated with compound 48/80 and this inhibition was reversed by the histamine antagonists (Masini et al., 1982).

H3R and H4R have about 40% sequence homology and are functionally related (Morse et al., 2001; Zhu et al., 2001). Both of them are activated by α-methylhistamine (H3/4R agonist) and inhibited by thioperamide (antagonist). Mouse MC were reported to express H4R and to lack H3R expression (except brain MC that do express H3R; Pillot et al., 2002; Gibbs and Levi-Schaffer, 2012). In mouse MC, the H4R has been shown to couple to Ca^2+^ mobilization, but not to cAMP, in a Ptx-sensitive manner (Hofstra et al., 2003; Rosethorne and Charlton, 2011). H4R in mouse MC affects chemotaxis and intracellular calcium mobilization, and although it is not involved in their degranulation, the receptor activation and subsequent histamine release leads to recruitment of effector cells, especially eosinophils, rich in H4R to the site of chronic allergic inflammation (Hofstra et al., 2003). H4R activation of murine MC increases leukotriene B4 (LTB4) release and supports neutrophil recruitment induced by zymosan (Takeshita et al., 2003). H4R blockade was shown to decrease MC and eosinophil migration to the airway epithelial tissue after guinea pigs allergen exposure (Yu et al., 2008). Blocking the H4R also decreases the MC migration in presence of CXCL12 *in vitro* (Godot et al., 2007). Therefore, H4R plays a role in allergy by mediating the recruitment of cells to the site of allergic inflammation and also by controlling this last effect together with H1R. It has recently been demonstrated that in a mouse model of allergic pruritus, the H4R antagonist JNJ7777120 is more effective in reducing the response to histamine release than H1R antagonist (Dunford et al., 2007). It is foreseen that combinatorial therapy with both H4R antagonists – when available for human use – to target mostly MC and eosinophils and H1R antagonists could be a potent new therapy for some allergic responses rather than just H1R antagonists.

### Serotonin receptors (5HTRs)

Serotonin is a neurotransmitter that can modulate a number of functions also outside the neural system (Berger et al., 2009). Serotonin receptors, also known as 5-hydroxytryptamine receptors (*5HTRs*), are classified in humans into seven main families with an additional subtype for two of the families (IUPHAR Receptor Database, 2008). Although 5HTRs have been known for a long time they have only recently been characterized on MC. With the exception of the 5HTR3, a ligand gated ion channel, all other serotonin receptors are GPCRs (Ritter et al., 2012) that activate an intracellular second messenger cascade to produce an excitatory or inhibitory response.

5HT1AR is negatively coupled to adenyl cyclase via Gi and it is expressed on hMC (Kushnir-Sukhov et al., 2006; Ritter et al., 2012) and mBMMC (Kushnir-Sukhov et al., 2006). It is expressed on MC in normal skin and its activation induces inflammation, due to MC migration and adherence in the site of inflammation, but it does not involve degranulation (Nordlind et al., 2008). Even though a still controversial issue, hMC were found to be able to secrete serotonin both in IgE-dependent and independent manner (Askenase et al., 1991; Matsuda et al., 1995). This was further shown to activate effector T cells and macrophages (Young and Matthews, 1995), and also could show the possibility of an autocrine regulation of the MC. 5HT/5HT1AR are involved in allergic contact eczema, atopic eczema, psoriasis (Nordlind et al., 2008), and also expressed in human mastocytosis (Ritter et al., 2012).

## Purinergic Receptors and ATP-Hydrolyzing Enzymes

### Purinergic receptors

*Purinergic receptors*, also known as purinoceptors, are a family of plasma membrane molecules involved in several cellular functions such as vascular reactivity, apoptosis, and cytokines secretion. The term, *purinergic receptor* was originally introduced to illustrate specific classes of membrane receptors that mediate relaxation of gut smooth muscle as a response to the release of Adenosine 5′-triphosphate (ATP) – P2R receptors or adenosine – P1R (King and Burnstock, 2002). There are three known distinct classes of purinergic receptors, referred to as P1, P2X, and P2Y receptors comprehending different subtypes (Bulanova and Bulfone-Paus, 2010). P2Y receptors, in addition to ATP, respond also to different nucleotides (ADP, UDP, UTP, UDP, and UDP-glucose). P1 and P2Y are GPCRs whereas P2X is a ligand gated ion channel.

Mast cells from most species express P1Rs: A_1_, A_2A_, A_2B_, and A_3_ adenosine receptors (Hua et al., 2011). Additionally they express a large variety of P2R. hCBMC, for example, express P2X_1_, P2X_4_, P2X_7_, P2Y_1_, P2Y_2_ (Bulanova and Bulfone-Paus, 2010), P2Y_12_, P2Y_13_, and P2Y_11_ (Feng et al., 2004), while mBMMC (derived from C57BL/6 mice) express the mRNA of P2X_1–4_, P2X_6–7_, and all P2Y receptors with the exception of P2Y_2_. P815 mastocytoma cells (derived from DBA/2 mice) express mRNA for all P2X and P2Y receptors (Bulanova and Bulfone-Paus, 2010). Activation of P2XR leads to the formation of a non-selective cationic channel (Burnstock, 2007), that triggers the activation of a number of intracellular signaling molecules, including MAPK, which is connected to cytokine secretion and release. ATP induces purinergic receptor P2X_7_ mediated membrane permeabilization, apoptosis, and cytokine expression in murine MC (Bulanova et al., 2009).

Stimulation of P2YR generally leads to phospholipase C (PLC) activation that cleaves PI(4,5)P2 to form IP3 and diacylglycerol, an activator of protein kinase C (PKC). Only the P2Y_11_ receptor is directly coupled to activation of adenylate cyclase (AC) and PLC, while P2Y_12_, P2Y_13_, and P2Y_14_ receptors negatively affect cAMP synthesis (Van Kolen and Slegers, 2006). Extracellular nucleotides activating P2 receptors are involved in the regulation of MC degranulation. P2Y_13_ receptor in a rat MC line (RBL-2H3) activation by ADP leads to intracellular calcium mobilization and release of β-hexosaminidase. Nucleotides via P2Y_2_ mediate chemotaxis in mBMMC and CD34+ progenitors (Bulanova and Bulfone-Paus, 2010).

ATP acts as an autocrine and paracrine factor that enables the intercellular communication. MC IgE-dependent degranulation induces the release of ATP that influences the neural cells via P2X and P2Y receptors (Bulanova and Bulfone-Paus, 2010).

A study recently reported by Yip et al. showed a biphasic effect of adenosine in hCBMCs, implicating the A_1_R in potentiation, and A_2_BR receptor in the inhibition of anti-IgE-induced degranulation (Yip et al., 2009; Hua et al., 2011). Interestingly, also leukotriene E4 acts as ligand for P2Y_12_ in LAD-2 cells. In addition it can induce purinergic receptor mediated pulmonary inflammation in mice (Paruchuri et al., 2009). It has been shown that adenosine binding to P1R on airway smooth muscle, goblet cells, MC, and neurons contributes to the pathogenesis of asthma (Okayama et al., 2008; Vieira et al., 2011). Adenosine inhibits and potentiates IgE-dependent histamine release from human lung MCs by an A_2_-purinoceptor mediated mechanism (Hughes et al., 1984).

Xanthine derived drugs such as theophylline that have been widely used for asthma, have been found to be both antagonists/agonists of adenosine receptors and phosphodiesterase inhibitors on MC. Several antagonists are currently available for research use, such as suramine (Gever et al., 2006). Although the purinergic receptors are well described in the nervous system, their discovery on MC is still relatively new and further research is required in order to understand their specific function and effects on these cells.

### CD203c

*CD203c* (E-NPP3) belongs to a family of ecto-nucleotide pyrophosphatase/phosphodiesterases (E-NPPs) that catalyzes the cleavage of phosphodiester and phosphosulfate bonds of a variety of molecules, including deoxynucleotides NAD and nucleotide sugars (Buhring et al., 2004). These type II TM proteins are composed of a short N-terminal cytoplasmic domain, followed by the TM region, two somatomedin-like domains, the catalytic domain and a C-terminal endonuclease-like domain. Recently, CD203c has been defined as a novel activation-linked surface antigen on MC that is upregulated in response to IgE receptor cross-linking and is overexpressed on neoplastic MC in patients with systemic mastocytosis (Hauswirth et al., 2008). CD203c on basophils serves as a marker to diagnose various allergic diseases such as asthma (Ono et al., 2010). The expression and role of ATP-hydrolyzing enzymes for the biology of MC is still under investigation.

## Corticotropin-Releasing Hormone and Endocannabinoids

Even though Corticotropin-releasing hormone (CRH) and endocannabinoids receptors have been typically described as central nervous system (CNS) receptors involved in regulation of the hypothalamic–pituitary–adrenocortical axis and in drugs response (Goeders and Guerin, 2000; Steiner and Wotjak, 2008), these receptors have also been found to be expressed on hematopoietic cells thus regulating peripheral conditions.

### Corticotricotropin releasing hormone receptors

Corticotricotropin releasing hormone receptors (*CRHRs*) are GPCRs which are activated by urocortin (UCN), CRH, or substance P and consist of two receptors-CRH-R1 and CRH-R2, each encoded by a separate gene (Theoharides et al., 1998; Slominski et al., 2001; Asadi et al., 2012). CRHR signaling involves cAMP/PKA pathway or alternatively MAPK or intracellular calcium (Ca^2+^)/PKC pathways, depending on the site of activation (Grammatopoulos, 2011).

CRH-R1 is sensitive to β-arrestin-dependent signaling desensitization and internalization. Receptor endocytosis is also one of the mechanisms employed by CRH-R1 to induce ERK1/2 and p38 MAPK phosphorylation and activation intermediates (Markovic et al., 2008). CRH-R1 variant is expressed in a tissue-specific manner; it is present in anterior pituitary as well as in peripheral sites such as reproductive tissues (myometrium, endometrium, and chorion trophoblast cells) and MC (Nezi et al., 2010).

Corticotropin-releasing hormone is secreted under stress and activates the hypothalamic–pituitary–adrenal axis. Interestingly a similar network is found in the skin (Asadi et al., 2012). Addition of CRH to the LAD-2 (leukemia MC line cells) primed with substance P induces synthesis and release of IL-8, TNF, and vascular endothelial growth factor (VEGF) 24 h later (Asadi et al., 2012). MC activation by CRH induces gene expression of neurokinin (NK-1). The ability of CRH to activate MC may explain its pro-inflammatory actions and the pathophysiology of certain skin conditions, which are precipitated or exacerbated by stress, such as atopic dermatitis, eczema, psoriasis, and urticaria (Theoharides et al., 1998). CRH has pro-inflammatory actions not only on its own, but also by augmenting the expression of the respective receptors on human skin MC (Asadi et al., 2012).

### Endocannabinoids receptors

The *cannabinoid receptors* are a class of cell membrane receptors belonging to the GPCRs superfamily. They contain seven TM spanning domains and are divided into two subtypes, termed cannabinoid 1 (CB1) and cannabinoid 2 (CB2). The endocannabinoid system has been recognized as a major neuromodulatory system, which functions to maintain brain homeostasis (Steiner and Wotjak, 2008). The CB1 receptor is expressed mainly in the brain CNS, but also in the lungs, liver, and kidneys. The CB2 receptor is expressed mainly in the immune system and in hematopoietic cells (Pacher and Mechoulam, 2011).

*CB1* was found to be expressed on human skin MC (Sugawara et al., 2012) and be activated by endocannabinoids such as anandamide (AEA) and 2-arachidonoylglycerol. CB1 and CB2 receptors can induce inhibition of adenylate cyclase activity and phosphorylation with activation of p42/p44 MAPK, p38 MAPK, and JNK as signaling pathways to regulate nuclear transcription factors. The CB1 receptor regulates K^+^ and Ca^2+^ ion channels, probably via Go/i (Howlett, 2005). A common role of CB1 and CB2 receptors appears to be the modulation of ongoing release of chemical messengers. The activation of CB2 receptors on MC has direct anti-inflammatory effects due to a decrease in mediators released from the cells (Pini et al., 2012).

The best CB2-selective agonists that have been developed so far include L-759633, L-759656, and JWH-133, all structural analogs of D9-THC. Other notable examples are the non-classical cannabinoid, HU-308, and the aminoalkylindole, AM1241 (Pertwee, 2006). The activation of CB1 receptor on bronchial nerve ending has a bronchodilatory effect (Pini et al., 2012). However, inhibiting the enzymatic inactivation of pharmacologically active endogenous molecules that do not serve as endocannabinoids or causing an accumulation of endocannabinoid molecules at non-CB1, non-CB2 targets such as the TRPV1 receptor or the putative abnormal-cannabidiol receptor, might lead to adverse effects.

Endocannabinoids limit excessive MC maturation and activation in human skin *in situ* and regulate SCF expression, by increasing its production in human hair follicle epithelium via CB1 stimulation (Sugawara et al., 2012). On the other hand in the ovalbumin induced lung sensitization model in CB1 and CB2 deficient mice a reduction in IgE production and attenuation of bronchoalveolar lavage fluid neutrophilia was described (Kaplan et al., 2010). In some studies, activation of CB1R seems to inhibit the allergic inflammation, while in others it is still controversial. Either way, we can conclude that CB1R activation is a potential target to be used in the treatment of allergy, although further research is still required.

## MC Receptors Cross-Talk

**Figure 1:**
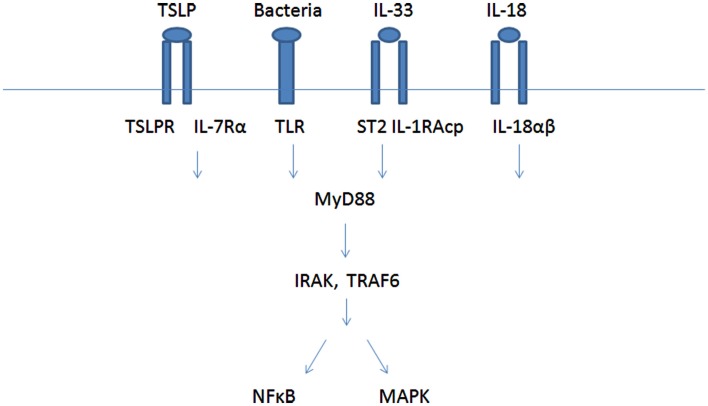
**Mast cells co-activation by IL-18, IL-33, TSLP, and TLR receptors**.

Cross-talk between receptors might result in co-activation or in inhibition of cell response. Co-stimulation is often required for the development of an effective immune response, and has been recognized to participate in antigen-specific signal from lymphocytes antigen receptors. In the last few years, parallel to the identification of the new MC receptors, some cross-talk effects between receptors were described, resulting either in co-activation (synergism or additive effects) or in inhibition. Most of the works have dealt with the FcεRI mediated MC activation. For example IL-18R, IL-33R, TSLP, and TLRs were found to work via the same signaling pathway which activates MyD88, IRAK, TRAF, and MAPK and NF-kB (Figure 1). GPCRs on MC can also co-interact to produce different responses.

### MC co-stimulation

Due to co-stimulation and cross-talk significance in modulation of MC activity, we chose to review some recent examples of this phenomenon. CCRs are known stimulatory and co-stimulatory molecules expressed on MC. Fifadara et al. showed for the first time the formation of cytoneme-like cellular extensions by BMMC, a totally new feature of these cells, upon co-stimulation of FcεRI and CCR1 with antigen and CCR1 ligand, macrophage inflammatory protein-1α. This co-stimulation was more effective in some morphology changes and mediator release than FcεRI stimulation alone (Fifadara et al., 2010). Thus, CCR can contribute to the inflammatory and allergic responses by formation of cytonemes, as a way to communicate between MC and other cells. Recently, the same group has identified 32 genes that were differentially regulated by a co-stimulation of antigen and the CCR1 ligand CCL3, compared to stimulation with antigen or CCL3 alone, in RBL-CCR1 transfected cells. Four genes were mostly up regulated 3 h post co-stimulation (*Ccl7*, *Rgs1*, *Emp1*, *RT1-S3*), but only the CCL7 protein was expressed at a higher level 24 h following co-stimulation. Among chemokines and cytokines tested, only CCL2 protein showed higher expression levels and IL-6 was seen only after co-stimulation, although in a very low level (Aye et al., 2012). The authors suggested therefore that CCL2, CCL7, and IL-6 may be important for MC regulation in the late phase of the allergic response.

Another novel example of co-stimulation is the one between FcεRI and adenosine receptor. Nunomura et al. showed that co-stimulation of a low antigen dose together with adenosine can induce MC degranulation in a synergistic way, through cooperation of the two receptors mediated signaling. This was suggested to be relevant to the immediate asthma response upon bronchial challenge with low-dose allergen (Nunomura et al., 2010). It has also been shown that mBMMC mutant in the FcεRIβ-chain-ITAM could not be degranulated upon co-stimulation, through PI3K-signaling pathway (Nunomura et al., 2010). These findings can provide more detailed insight to which and how specific parts of the β chain and the PI3K-signaling are important for MC co-activation.

In mouse MC, thrombin, a serine protease, by binding to the protease-activated receptor (PAR)-1 TRAP(14) induces the secretion of IL-6 in a dose dependent fashion, but not of TNFα. Co-stimulation of low level thrombin with an allergen, can synergistically enhance IL-6 secretion through FcεRI signaling and the PI(3) and sphingosine-kinase pathways (Gordon et al., 2000).

Drube et al. reported a cross-activation of IL-33R and c-kit receptor in human and murine MC, after joining of IL-33R upon ligand binding, to a constitutively bound complex composed of c-kit and IL-1R accessory protein. In primary MC, the c-kit ligand SCF, is necessary for IL-33 induced cytokine production (Drube et al., 2010). Silver et al. showed that IL-33 in HMC-1 cells induced a synergistic activation effect together with adenosine, C5a, SCF, and NGF receptors. In primary hMC, IL-33R was also synergized with FcεRI activation (Silver et al., 2010).

CD48 was found to be a co-stimulatory receptor for inducing different effects in various hematopoietic cells by binding several ligands (Elishmereni and Levi-Schaffer, 2011). We have recently found that hCBMC and eosinophils can be directly activated by CD48 ligand/s (Elishmereni et al., 2011). In addition we detected some co-stimulatory effects when CBMC were activated by IgE-dependent mechanisms (Minai-Fleminger, Y., Levi-Schaffer, F., unpublished data).

CD84 is a self binding receptor from the signaling lymphocyte activating molecules (SLAM) family. However it was shown on transfected RBL-2H3 cells to inhibit FcεRI degranulation (Oliver-Vila et al., 2008). Additionally CD84 was found to be expressed on hMC (Alvarez-Errico et al., 2011), and shown to be tyrosine phosphorylated upon co-stimulation with FcεRI. This led to reduction in granule release and in the release of IL-8 and GM-CSF in LAD-2 cells and human CD34+ derived MC (Alvarez-Errico et al., 2011).

As reported in the past by our group, MC express DNAX accessory molecule 1 (DNAM-1, CD226) and eosinophils express its ligand Nectin-2 (CD112). It was shown that CD226 engagement with FcεRI induces an activating synergistic effect on MC and blocking of the ligand CD112 reduced the hyperactivity caused by IgE-dependent MC activation (Bachelet et al., 2006). To conclude, these few examples of co-stimulatory responses on MC can possibly help in understanding and foreseeing the complex allergic inflammatory response and be essential for the development of new anti-allergic therapy.

### MC inhibitory receptors and MC inhibition

Inhibitory receptors classically described on NK (Cantoni et al., 1999; for review, see Pegram et al., 2011) comprise two main families of the Ig receptor super-family and the c-type (calcium dependent) lectin superfamily. On MC, FcγRIIB, CD300, CD72, and sialic acid binding Ig-like lectins (Siglec)8, 7, etc., have been described (Karra and Levi-Schaffer, 2011). Since the FcγRIIB receptor has been extensively investigated (see Daeron et al., 2008; Bruhns et al., 2009) and we discussed some receptors bringing about MC inhibition, such as CB1, under their respective families, here we will consider only the most newly described typical IRs.

**Figure 2:**
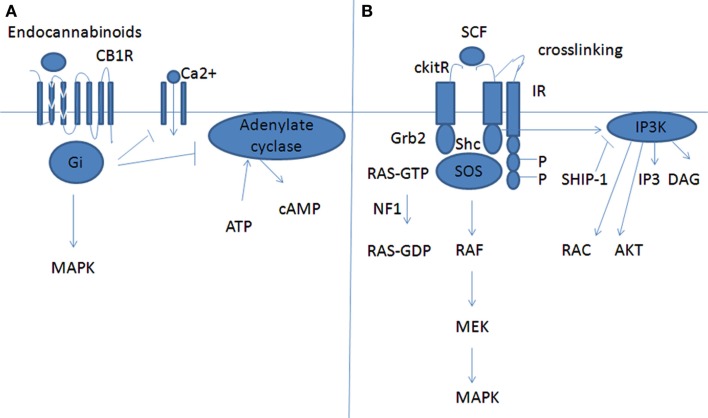
**Inhibition of MC maturation by CB1 or by a classical IR receptor**. **(A)** CB1 inhibition effect. **(B)** Inhibition of c-kit mediated signaling by an inhibitory receptor (IR). Downstream molecules to CB1 or to c-kit cross-linked with the inhibitory receptor (cross-linking is mandatory to inhibitory effect).

Inhibitory receptors expressed on MC can modulate their function by inhibiting typically the signaling from receptors associated with tyrosine kinases, i.e., FcεRI and c-kit (Figure 2). IRs contain immunoreceptor tyrosine based inhibition motifs (ITIMs) that down regulate the activation signals transmitted through immunoreceptor tyrosine based ligand ITAMs. The engagement of IRs suppresses cell activation by promoting dephosphorylation reactions. Upon activation of ITIM-containing receptors, tyrosine residues within the motifs become phosphorylated. This leads to the recruitment of protein phosphatases, Src homology 2 (SH) domain containing protein tyrosine phosphatase-1/2 (SHP-1 and SHP-2) and lipid phosphatases SH2 domain bearing inositol phosphatase (SHIP1). SHP-1/2 inhibits the action of tyrosine kinase, while SHIP1 terminates the PI3K-mediated pathway (Karra and Levi-Schaffer, 2011). Herewith we are going to illustrate the CD300a, Siglec 7 and 8, and CD72 IRs.

The CD300 family of myeloid immunoglobulin receptors includes activating (CD300b, CD300e) and inhibitory (*CD300a*, CD300f) members, as well as molecules of uncertain functions presenting a negative charge within their TM domain (CD300c, CD300d; Martinez-Barriocanal et al., 2010). None of the activating receptors identified contains a charged glutamic acid residue in the TM that is present in CD300c, suggesting that CD300c might deliver activating signals via signaling proteins other than those previously observed or that CD300c is not an activating receptor (Lankry et al., 2010). The function of CD300d is related to the regulation of the expression of other CD300 molecules and the composition of CD300 complexes on the cell surface (Comas-Casellas et al., 2012).

The CD300 IR are type I TM glycoproteins with a single IgV-like extracellular domain and a membrane proximal region, a TM region, and a cytoplasmic region with ITIM motif (Lankry et al., 2010). The cytoplasmic domains of CD300a and CD300f contain internalization motifs and the cell surface expression of these molecules might be regulated, in part, by internalization (Clark et al., 2009). The expression of CD300a and CD300f was shown in MC (Clark et al., 2009), where CD300a presents on MC from nasal polyps, lungs, and on hCBMC (Bachelet et al., 2005) and its mouse ortholog LMIR-1, is expressed on mBMMC (Kumagai et al., 2003). CD300a is also expressed on NK, T cell subsets, neutrophils, eosinophils, monocytes DC, and more recently it has been characterized on basophils (Cantoni et al., 1999; Bachelet et al., 2005; Munitz et al., 2006a; Alvarez et al., 2008; Sabato et al., 2012).

It has been recently reported that CD300a interacts with polar lipids, including the major phospholipids of cell membranes, and is able to transduce intracellular signals after lipid binding. One of CD300a ligands is phosphatidylserine which directly binds CD300a and phosphorylates ITIM in the cytoplasmic portion of CD300a on BMMC (Nakahashi-Oda et al., 2012). Interestingly, Simhadri showed that CD300a phosphatidylethanolamine and phosphatidylserine bind to CD300a, and modulate the phagocytosis of dead cells. CD300a down regulates the uptake of apoptotic cells by macrophages and its ectopic expression in CD300a-negative cell lines also decreased the engulfment of dead cells (Simhadri et al., 2012). Using a co-transfection assay on THP-1 cells, Kim et al. (2012) showed that while CD300a blocked only MyD88 induced events, CD300f blocked both MyD88 and TRIF.

CD300a reduced survival of MC and eosinophils by decreasing the effect of c-kit on MC, and the IL-5, GM-CSF effect on eosinophils (Bachelet et al., 2005, 2008). Moreover a bi-specific Ab to CD300a and IgE inhibited acute airway inflammation in an experimental asthma mouse model and passive cutaneous anaphylaxis (Bachelet et al., 2008). Also a bi-specific Ab against CD300a and CCR3 inhibited chronic airway inflammation in murine asthma, possibly by its binding on both MC and eosinophils and their consequent inhibition (Munitz et al., 2006b). Disease-wise there is evidence for alterations in the expression and function of the CD300 family in patients with psoriasis (Clark et al., 2009).

*Siglec-8* appears on hMCs at the same time as other MC markers such as FcεRI (Karra et al., 2009). When cross-linked by mAbs on hpbMC, Siglec-8 does not lead to apoptosis as it does on eosinophils, but rather to strong inhibition of histamine and prostaglandin D2 secretion and of [Ca^2+^] influx (Yokoi et al., 2008; Bochner, 2009; Karra et al., 2009). Siglec-8 is known to specifically recognize the sialoside sequence 6′-sulfo-sLex (Tateno et al., 2005). Siglec-8 has been shown to be associated with asthma (Gao et al., 2010). We have found *Siglec 7*, another lectin-binding IR, that was previously described on eosinophils (Munitz et al., 2006a) to be expressed and functional on hMC (Mizrahi, S., Karra, L., Ben Zimra, M., and Levi-Schaffer, F., unpublished data).

*CD72* (Lyb-2) is an ITIM-containing 45 kDa type II TM protein of the C-type lectin family (Wu and Bondada, 2009) whose natural ligands have been identified as CD100 or Semaphorin 4D (Sema4D). CD72, which is considered to be an important co-receptor regulating B-cell activation (Wu and Bondada, 2009), is also expressed on mouse NK cells (Alcon et al., 2009) and on hPBMC and human cell lines (Kataoka et al., 2010). Ligation of CD72 reduced SCF-mediated proliferation, chemotaxis, and MCP-1 (or CCL2) production in hMCs and the suppression of growth of HMC-1.2 harboring the gain-of-function mutation in KIT gene (Kataoka et al., 2010).

In conclusion IR, that have been shown to mostly modulate on MC the activation of FcεRI and c-kit, may have by themselves or by cooperation with other receptors, inhibitory functions on other receptors not necessarily linked to tyrosine kinases mechanism, although this still has to be thoroughly investigated.

## Conclusion

Mast cells express a variety of receptors that enable them, both in activating or inhibiting ways, to modulate immune response and can be used as treatment targets in disease conditions. While with some of the described receptors such as H4 and purinergic receptors, we are close to developing a drug, with others, such as TSLP, more basic studies on their functions are needed. Overall, evidences show that MC receptors have a strong therapeutic potential but still a better understanding of their pathophysiological roles, expression and their role in the immune system is required in order to develop effective and side effect free therapeutic interventions in the near future.

## Conflict of Interest Statement

The authors declare that the research was conducted in the absence of any commercial or financial relationships that could be construed as a potential conflict of interest.
